# 2451: Optimal study design for Diagnostic Accuracy Studies: Differential verification Versus partial verification

**DOI:** 10.1017/cts.2017.109

**Published:** 2018-05-10

**Authors:** Yizhe Xu, Joseph B. Stanford, Kristina Allen-Brady, Nan Hu

**Affiliations:** The University of Utah School of Medicine, Salt Lake City, UT, USA

## Abstract

OBJECTIVES/SPECIFIC AIMS: To compare the accuracy and precision for estimating the diagnostic accuracies (sensitivities and specificities) between differential verification (DV) and partial verification (PV) methods. Comparisons were made under scenarios with different values of design parameters including disease prevalence, proportion of verification for positive results, proportion of verification for negative result, sensitivity and specificity of the brass standard (BS) test in DV method. Through comparing 2 different verification methods under different scenarios, we give suggestions that which verification method is optimal under different design settings. METHODS/STUDY POPULATION: For both PV and DV methods, simulation studies were performed using statistical package R, version 3.1.3. We were primarily interested in studying how the unbiasedness and precision for estimation of diagnostic accuracies (sensitivity and specificity) of an index test change with the following design parameters: disease prevalence, proportion of verification for positive test results, the proportion of verification for negative test results, and the sensitivity and specificity of a BS test. We chose different values for each of the above parameters. For each estimation, we allowed values in only 1 parameter to change by fixing the other 2 parameters, so that the effect of each design parameter on the unbiasedness and precision of both sensitivity and specificity can be determined. For the DV method, we also developed an analytical method to estimate the sensitivity and specificity of an index test using a quadratic equation with a unique solution of the specificity and sensitivity. RESULTS/ANTICIPATED RESULTS: For rare disease with prevalence less than 1%, the PV method resulted in a less biased and more precise estimate of sensitivities and specificities of the index test. If the disease prevalence was between 1% and 10%, the DV method using a BS test with moderate or high sensitivity and specificity (sensitivity and specificity >90%) resulted in a less biased and more precise estimate of diagnostic accuracies of the index test. When the disease prevalence was greater than 10%, the PV method was superior when the BS test had sensitivity and specificity <80%, and the DV method was superior when the BS test had both sensitivity and specificity >90%. When the proportion of verification of positive test results was <30% or >70%, the DV method yielded smaller bias for the estimated specificity than the PV method. However, the PV method generated a much smaller mean square error (MSE) for specificity than the DV method when the proportion of verification for positive test results was >50%. Although the disease prevalence was >10% and the proportion of verification of positive test results was <30%, the DV method resulted in a smaller MSE for specificity. DISCUSSION/SIGNIFICANCE OF IMPACT: Disease prevalence and proportions of verification for patients with positive and negative test results influence the accuracy of a new diagnostic test. If a new index test for a very rare disease is evaluated, the PV method should be used for assessing the performance of the index test. When a disease prevalence is >1%, the DV method will result in a less biased and more precise estimate of diagnostic accuracy of an index test, if the BS test itself used in the DV method has large specificity and specificity. One concern of using BS test for the DV method is the clinical cost. Depending on the disease type, the BS tests usually are imperfect, but may be less aggressive and/or less expensive than the gold standard test. Moreover, as all clinical examinations require professional personnel to perform, verification of the index test for relative large proportion of a large cohort of patients could become a burden on human resources. Thus, the future research of the optimal design method for a diagnostic accuracy study should be based on the comprehensive cost-effectiveness analysis.

